# Anti-Drug Antibodies in Patients with Fabry Disease Treated with Agalsidase-Alpha or -Beta: A Retrospective Italian Multicenter Study

**DOI:** 10.3390/cells15212015

**Published:** 2026-11-05

**Authors:** Andrea Matucci, Michael Diomaiuti, Amelia Morrone, Pasquale Esposito, Federico Pieruzzi, Maurizio Pieroni, Emanuele Vivarelli, Elena Biagini, Raffaello Ditaranto, Maria Alessandra Schiavo, Calogero Lino Cirami, Leonardo Caroti, Egrina Dervishi, Ilaria Romani, Cristina Sarti, Mario Mangeri, Barbara Trezzi, Letizia Roggero, Claudia Sgattoni, Alessia Gennarini, Giancarlo la Marca, Giulia Forni, Giacomo Mascia, Marina Rinaldi, Annamaria Del Franco, Alessia Argirò, Sandro Feriozzi, Matteo Accinno, Alessandra Vultaggio

**Affiliations:** 1 Immunoallergology Unit, Careggi University Hospital, 50134 Florence, Italy; andrea.matucci@unifi.it (A.M.); michael.diomaiuti@unifi.it (M.D.); emanuele.vivarelli@unifi.it (E.V.); alessandra.vultaggio@unifi.it (A.V.); 2 Laboratory of Molecular Genetics of Neurometabolic Diseases, Neuroscience and Human Genetics Department, Meyer Children’s Hospital IRCCS, 50134 Florence, Italy; amelia.morrone@meyer.it (A.M.); marina.rinaldi@meyer.it (M.R.); 3 Department of Neurosciences, Psychology, Drug Research and Child Health (NEUROFARBA), University of Florence, 50139 Florence, Italy; 4 Unit of Nephrology, Dialysis and Transplantation, AOM-IRCCS Ospedale Policlinico San Martino, 16132 Genova, Italy; pasquale.esposito@unige.it; 5 Department of Internal Medicine and Medical Specialties (DIMI), University of Genoa, 16132 Genova, Italy; 6 Nephrology, Fondazione IRCCS San Gerardo dei Tintori, 20900 Monza, Italy; federico.pieruzzi@unimib.it (F.P.); barbara.trezzi@irccs-sangerardo.it (B.T.); letizia.roggero@irccs-sangerardo.it (L.R.); 7 School of Medicine and Surgery, University of Milano-Bicocca, 20126 Milan, Italy; 8 Department of Experimental and Clinical Medicine, University of Florence, 50134 Florence, Italy; maurizio.pieroni@unifi.it (M.P.); matteo.accinno@unifi.it (M.A.); 9 Cardiomyopathy Unit, Careggi University Hospital, 50134 Florence, Italy; delfrancoa@aou-careggi.toscana.it (A.D.F.); argiroa@aou-careggi.toscana.it (A.A.); 10 Cardiology Unit, IRCCS Azienda Ospedaliero-Universitaria di Bologna, Policlinico di Sant’Orsola, 40138 Bologna, Italy; elena.biagini73@gmail.com (E.B.); raffaello.ditaranto@studio.unibo.it (R.D.); maria.schiavo2@studio.unibo.it (M.A.S.); 11 European Reference Network for Rare, Low Prevalence, and Complex Diseases of the Heart (ERN GUARD-Heart), 40138 Bologna, Italy; 12 Nephrology, Dialysis and Transplantation Unit, Careggi University Hospital, 50134 Florence, Italy; lino.cirami56@gmail.com (C.L.C.); carotile@aou-careggi.toscana.it (L.C.); dervishie@aou-careggi.toscana.it (E.D.); 13 NEUROFARBA Department, University of Florence, 50019 Florence, Italycristina.sarti@unifi.it (C.S.); 14 Stroke Unit, Careggi University Hospital, 50134 Florence, Italy; 15 Nephrology and Dialysis Department, Belcolle Hospital, 01100 Viterbo, Italy; mario.mangeri@asl.vt.it; 16 SOSD Genetica Medica e Coordinamento Malattie Rare, Azienda Ospedaliero-Universitaria delle Marche, 60126 Ancona, Italy; claudia.sgattoni@ospedaliriuniti.marche.it; 17 Unit of Nephrology, Azienda Socio-Sanitaria Territoriale (ASST) Papa Giovanni XXIII, 24127 Bergamo, Italy; agennarini@asst-pg23.it; 18 Newborn Screening, Clinical Biochemistry and Clinical Pharmacy Lab Meyer Children’s Hospital IRCCS, 50139 Florence, Italy; giancarlo.lamarca@meyer.it (G.l.M.); giulia.forni@meyer.it (G.F.); 19 Department of Experimental and Clinical Biomedical Sciences, University of Florence, 50134 Florence, Italy; 20 Divisione di Nefrologia e Dialisi Azienda Ospedaliera “G. Brotzu”, 09134 Cagliari, Italy; giacomo.mascia@aob.it; 21 Fondazione Policlinico Universitario Campus Bio-Medico, 00128 Rome, Italy

**Keywords:** anti-drug antibodies, enzyme replacement therapy, Fabry disease, predicted GLA null-type variants, immunogenicity, lyso-Gb3, α-Gal A activity

## Abstract

**Highlights:**

**What are the main findings?**

- A high prevalence of anti-drug antibodies was observed in patients receiving ERT, with no statistically significant difference between agalsidase-α and agalsidase-β.
- Male sex and predicted null-type *GLA* variants were independently associated with anti-drug antibodies in patients with Fabry disease receiving enzyme replacement therapy, whereas a higher residual α-Gal A activity at diagnosis was associated with lower odds of it.

**What are the implications of the main findings?**

- Genotype- and sex-informed risk stratification may help identify patients who warrant closer immunogenicity monitoring during enzyme replacement therapy.
- The role of ADAs in risk stratification is promising but needs further validation in wider cohorts of patients.

**Abstract:**

(1) Background: Anti-drug antibodies (ADAs) may develop during enzyme replacement therapy (ERT) for Fabry disease and potentially affect biochemical response. Evidence have been derived mainly from males, while comparisons between agalsidase-alpha and agalsidase-beta remain limited by differences in ADA assays. The aim of the study is to evaluate ADA prevalence and titers in patients with Fabry disease treated with agalsidase-alpha or agalsidase-beta and to investigate associations with sex, GLA genotype, residual α-galactosidase A activity, and lyso-Gb3 levels. (2) Methods: This multicenter observational study included 117 patients with Fabry disease receiving ERT (50 males, 67 females), treated with agalsidase-alpha (n = 52) or agalsidase-beta (n = 65). ADAs were measured using an in-house enzyme-linked immunosorbent assay, and lyso-Gb3 by liquid chromatography–tandem mass spectrometry. GLA variants were centrally classified as predicted null-type or predicted non-null-type on the basis of the variant class, and α-galactosidase A activity at diagnosis was normalized to the lower limit of the assay reference interval. Multivariable regression analysis identified factors associated with ADA positivity and lyso-Gb3 levels. (3) Results: ADAs were detected in 55/117 patients. Positivity was more frequent in males than females, with no statistically significant difference between agalsidase-alpha and agalsidase-beta. Predicted null-type variants were associated with higher ADA positivity and titers, particularly in males. In multivariable models, male sex and predicted null-type variants independently predicted ADA positivity, whereas higher normalized α-galactosidase A activity was associated with lower odds. ADA positivity was not independently associated with lyso-Gb3. (4) Conclusions: ADA detection was primarily associated with male sex and predicted null-type GLA variants; no statistically significant association with the agalsidase administered was found. The high prevalence of binding ADAs in females warrants confirmation using orthogonal and functional assays.

## 1. Introduction

Fabry disease (FD) is a rare, X-linked, lysosomal storage disease caused by pathogenic variants in the galactosidase alpha (GLA) gene leading to absent or reduced activity of α-galactosidase A (α-Gal A). The direct consequence is a systemic intracellular accumulation of the neutral glycosphingolipid globotriaosylceramide (Gb3) and of its deacylated derivative globotriaosylsphingosine (Lyso-Gb3) [1,2]. The intracellular accumulation of Gb3 causes an activation of inflammatory processes with progressive multi-organ damage which is clinically evident mainly at the level of the heart, kidneys and nervous system [3,4]. Male patients with the classic phenotype have little to no α-Gal A activity and typically develop early-onset symptoms while non-classic males who have residual α-Gal A activity generally display milder and late-onset manifestations [5]. On the other hand, female patients who are heterozygous for pathogenic GLA variants can display a broad spectrum of symptoms or remain asymptomatic or pauci-symptomatic [6].

Enzyme replacement therapy (ERT) based on the use of agalsidase-α, produced in a human cell line, and agalsidase-β, prepared by means of Chinese hamster ovary (CHO) cells, has been the standard treatment for FD, alongside new molecules that have enriched the available therapeutic armamentarium and others that are being tested to expand it further [7]. Recently, a PEGylated [polyethylene glycol (PEG)] molecule produced using tobacco plant cells to obtain a covalently cross-linked form of α-Gal A and designed to increase plasma half-life of the enzyme, named pegunigalsidase-α, had been approved [5,8].

A growing body of clinical and experimental evidence shows that a significant proportion of male patients with classical FD develop anti-drug antibodies (ADAs) during treatment with ERT, even though ADAs may also be detected in females and non-classical patients with milder forms of the disease [9]. The main reason why ADAs are detected particularly in classical male patients is that these subjects, due to their genetic defect, have little to no native enzymes, and therefore the immune system can react to the exogenously administered compound as if it were a foreign molecule. In fact, data from the literature reported that the majority of females tested negative for ADAs likely due to their heterozygous status and residual enzyme activity [10,11]. Even though clinical implications of ADA formation are still not completely understood, ADAs are thought to be responsible for loss of response to ERT and/or to induce adverse infusion reactions [12]. Several studies have shown that male FD patients with established ADA titers showed higher disease severity scores, and an accelerated renal decline and heart disease progression compared to ADA-negative subjects [13]. Similarly, infusion reactions occur in ADA-positive patients in which IgG, and in some cases IgE isotypes, have been detected [14,15].

The aim of this study was to assess the ADA positivity in a case series of male and female patients suffering from FD treated with agalsidase-α or -β and to analyze the relationship between ADA, Lyso-Gb3 plasma levels, genotype and residual α-Gal A activity.

## 2. Materials and Methods

### 2.1. Patients and Study Design

One hundred and seventeen patients diagnosed with FD were enrolled in Italian specialized Fabry Centers, comprising 67 females and 50 males, with a median age of 54 (IQR: 21) years. Among these, 52 (44.4%) patients (20 males and 32 females) were treated with agalsidase-α (0.2 mg/Kg every 2 weeks), and 65 (55.6%) patients (30 males and 35 females) were on agalsidase-β (1 mg/Kg every 2 weeks). Eight patients who had previously received a different ERT product (three treated with agalsidase-α and five with -β) were excluded before the analysis, so that none of the enrolled patients had been switched between products. No interruption of treatment was recorded in any of the patients. For each patient, data were available on Lyso-Gb3 levels during treatment, GLA variant and baseline residual α-Gal A activity. Demographic and clinical characteristics are summarized in Table 1. In addition, all individual genetic characteristics of every patient are shown in Table S1.

**Table 1:** Demographic and clinical characteristics of patients with Fabry disease.

	Overall (n = 117)	Agalsidase Alpha (n = 52)	Agalsidase Beta (n = 65)	*p* Values
Age, years	54 (21)	55 (21.3)	53 (15)	0.63
Males/Females n	50/67	20/32	30/35	0.47
Treatment duration (months)	84 (110.9)	130 (177)	68.5 (96.7)	**<0.001**
Lyso Gb3 (nmol/L)	7.7 (2.1)	8.0 (10.7)	7.6 (8.5)	0.70
Mutation category				
- missense n ° (%)	71 (60.7)	34 (65.4)	37 (56.9)	0.45
- del./ins./frameshift n °(%)	21 (17.9)	11 (21.2)	10 (15.4)	0.47
- nonsense n ° (%)	14 (11.9)	3 (5.8)	11 (16.9)	0.09
- in-frame n ° (%)	5 (4.3)	1 (1.9)	4 (6.2)	0.38
- splicing n ° (%)	3 (2.6)	2 (3.8)	1 (1.5)	0.58
- start-loss n ° (%)	3 (2.6)	1 (1.9)	2 (3.1)	>0.999
Allele category				
- predicted null-type n ° (%)	41 (35.0)	17 (32.7%)	24 (36.9%)	0.7
- predicted non-null-type n ° (%)	76 (65.0)	35 (67.3%)	41 (63.1%)	0.7
Tissue involvement °				
- Heart n ° (%)	93 (79.5)	39 (75)	54 (83.1)	0.28
- Kidney n ° (%)	67 (57.3)	27 (51.9)	40 (61.5)	0.30
- Nervous system n ° (%)	55 (47)	24 (46.2)	31 (47.7)	0.87
- Other n ° (%)	37 (31.6)	20 (38.5)	17 (26.2)	0.16

*Note*: Data are expressed as median (IQR); *p* values are applied to agalsidase-α/agalsidase-β comparisons. ° Tissue involvement was defined as the presence of signs or symptoms associated with Fabry disease. Heart ECG/Echocardiogram/Magnetic resonance imaging (MRI). Kidney: albuminuria, reduced renal function; nervous system: central nervous system MRI, peripheral electromyography, pain. Other cornea verticillate, chronic fatigue, angiokeratoma, gastrointestinal symptoms, anhidrosis, “Fabry crisis”. Statistically significant *p* values are shown in bold.

Each patient underwent a blood draw immediately before a scheduled ERT administration (that is 14 days after the previous one) for the detection of ADAs and Lyso-Gb3 plasma levels. Blood was collected in 5 mL tubes and centrifuged at 600× *g* for 10 min. Serum was then frozen at −80 °C and thawed no more than once before analysis. Patient sera were used to develop an ELISA test to assess ADAs. To determine the cut-offs for ADA positivity, sera from 40 healthy donors (HD) with no prior drug exposure were used (see below).

Personal data were pseudonymized at the coordinating center and handled in accordance with Regulation (EU) 2016/679 [16] and with Italian Legislative Decree 196/2003 [17] as amended by Legislative Decree 101/2018 [18].

This is a study combining retrospective collection of clinical, genetic and biochemical data at baseline and cross-sectional measurement of serum ADA and Lyso-Gb3 plasma levels. GLA genotyping and the determination of residual α-Gal A activity had been performed in all patients at the time of diagnosis, as part of the routine diagnostic work-up, and were not repeated for the purposes of the present work; the corresponding results were retrieved from the clinical records of each center. The only determinations performed specifically for this study were the measurement of ADA and of plasma Lyso-Gb3 on the sample drawn before a scheduled infusion.

### 2.2. GLA Molecular Analysis

All patients had a molecular diagnosis of FD established before enrolment, in the genetics laboratory of the referring center. Genomic DNA was extracted from peripheral blood leucocytes and the seven coding exons of GLA, together with the flanking intronic regions, were analyzed by Sanger sequencing or by a targeted next-generation sequencing panel, according to the protocol in use at the genetics center that performed the analysis. For the present study, all variants were re-annotated centrally according to HGVS recommendations against reference transcript NM_000169.3, with protein numbering referring to the 429-residue precursor (NP_000160), which includes a 31-residue signal peptide, and were reclassified according to ACMG/AMP criteria.

Variants were grouped into six classes: nonsense, deletion/insertion/frameshift, canonical splice-site, start-loss, missense and in-frame deletion/delins. For the main genotype analysis these six classes were collapsed into two categories. Nonsense, deletion/insertion/frameshift, canonical splice-site and start-loss variants were grouped as predicted null-type (n = 41); that is, the classes in which translation of a full-length product is abolished. Missense and in-frame deletion/delins variants were grouped as predicted non-null-type (n = 76). In-frame deletion/delins variants were deliberately kept outside the predicted null-type category: although a shortened product is unlikely to pass endoplasmic-reticulum quality control, this has not been demonstrated for the individual variants carried by our patients. The assignment reflects what the variant class predicts about the expression of endogenous protein and is not a measurement of protein output: it was not verified at protein level in these patients, and the three canonical splice-site variants were assigned on the basis of their position, without confirmation at the RNA level, because no RNA or viable cells from the diagnostic work-up were available. The two categories should therefore be read as predicted classes throughout. The dichotomy is also conservative in the opposite direction, since missense variants may produce a conformationally unstable protein that is retained and degraded before reaching the lysosome [19], so that the predicted non-null-type group may include variants with very little mature enzymes.

### 2.3. Reagents

For the development of the ELISA test, drugs (agalsidase-α and agalsidase-β) and sera were diluted in phosphate-buffered saline (PBS; Thermo FisherScientific, Waltham, MA, USA). The blocking solution consisted of 10% bovine serum albumin (BSA; PAN-Biotech GmbH, Aidenbach, Germany) in PBS. For the washing steps, PBS was supplemented with polyoxiethylenesorbitan monolaurate (Tween 20; Sigma-Aldrich Chemie GmbH, Taufkirchen, Germany) at a 0.05% concentration. The secondary antibody was a horseradish peroxidase-conjugated rabbit anti-human IgG (Jackson ImmunoResearch Laboratories, Inc., West Grove, PA, USA). Tetramethylbenzidine (TMB) and the stop solution (H_2_SO_4_) were purchased from: Thermo Fisher Scientific, Waltham, MA, USA.

### 2.4. α-Gal A Activity Assay

Residual α-Gal A activity was not measured for the purposes of this study: it was retrieved from the diagnostic record of each patient, as determined at the time of diagnosis in the laboratory of the referring center. Matrix, analytical platform and reference interval therefore vary between patients, could not be harmonized a posteriori, and the determinations could not be repeated, since diagnostic samples are not retained for this purpose. Activity had been measured on dried blood spots [87 patients (74.4%)] or on peripheral blood leucocytes [30 patients (25.6%)]. On dried blood spots the enzyme activity was determined by liquid chromatography–tandem mass spectrometry according to previously reported standard protocols [20], and is expressed per volume of blood (µmol/L/h). On leucocytes, cells were separated from whole blood by dextran sedimentation, and the enzyme activity was measured in triplicate with a fluorogenic assay using 4-methylumbelliferyl-α-D-galactopyranoside in the presence of N-acetyl-D-galactosamine, added to inhibit α-N-acetylgalactosaminidase, which hydrolyzes the same artificial substrate [19,20]; activity is expressed per milligram of protein (nmol/mg protein/h), with protein quantified by the bicinchoninic acid (BCA) assay. The determinations were performed in 5 laboratories for dried blood spots and 2 laboratories for leucocytes, and for every patient Table S1 reports the matrix, the analytical method, the measured value, the reference interval of the assay used and the resulting normalized value, so that each measurement can be traced to the assay that generated it.

Residual α-Gal A activity is therefore reported throughout as a fold change relative to the lower limit of the reference interval of the assay actually used for each patient, which is the only form in which values obtained with different methods can be compared. Normalization places all patients on a common scale relative to the threshold that each laboratory considers diagnostic, but it does not make the two matrices biologically equivalent: it rescales them, and any difference in the dynamic range of the two assays close to the lower limit is not removed by this operation. The enzymatic variable is accordingly used here as an ordered indicator of residual activity rather than as an absolute quantitative measurement.

### 2.5. Lyso-Gb3 Quantification

To quantify Lyso-Gb3, 50 μL plasma samples were diluted with 200 μL of acetone 45%, methanol 45%, and water 10% in a 1.5 mL Eppendorf tube (Eppendorf, Milano, Italy), vortexed for 20 s and then centrifuged at 16.100× *g* to precipitate proteins. The extract was transferred to a 2 mL glass vial (Agilent Technologies, Santa Clara, CA, USA) and evaporated under nitrogen flow. The dried sample was reconstituted in 150 μL of acetonitrile 70%, water 30% with 0.1% formic acid and immediately analyzed by liquid chromatography/tandem mass spectrometry.

### 2.6. ADA Assays

For ADA quantification, plates were coated with 100 μL of ERT commercial drugs at a concentration of 1 μg/mL in PBS. Coated plates were incubated overnight at room temperature (RT) and covered. All washing steps were automated using Automated Microplate Washer, MW12A; Shenzhen Mindray Bio-Medical Electronics CO., Ltd., Shenzhen, China. The day after, the plate was washed 3 times with 250 μL of PBS + 0.05% Tween and 200 μL of 10% BSA (diluted in PBS) was added for blocking. After 2 h of incubation at RT, the plate was washed 5 times with 250 μL of PBS + 0.05% Tween. Subsequently, 100 μL of serially diluted sera (from 1:100 to 1:102,400, and 1:20) in PBS was added. After 1 h of incubation at RT, in an agitator (Thermo-Shaker, PST 60HL, Bioclass S.r.l., Pistoia, Italy) at 300 rpm, the plate was washed as before. Then, 100 μL of secondary antibody (Horseradish peroxidase-conjugated rabbit anti-human IgG) at a concentration of 150 ng/mL in PBS was added. After another hour of incubation at RT, the plate was washed as before. Next, 100 μL of TMB was added and, after incubation for 25 min at RT in the dark and the addition of 100 μL of stop solution, the plate was read at 450 nm in a spectrophotometer (Glomax Multi Detection System; Promega Corporation, Madison, WI, USA). For positivity cut-off analyses of every ERT drug, 40 sera from HD were used. ADA titer was determined as the reciprocal of the highest dilution factor whose optical density (OD) exceeded the cut-off determined on HD sera for the corresponding dilution (cut-off values are given in Table 2). Sera with optical density lower than the 1:100 cut-off were considered ADA-negative. Sera that maintained positivity at the highest dilution (1:102,400) were further diluted 1:2 and the ADA titer was re-tested. The 1:20 dilution was used as a confirmatory test for positivity.

**Table 2:** Mean optical density values and cut-off values determined in 40 healthy controls using serial dilutions.

	Agalsidase Alpha			Agalsidase Beta		
Serum Dilution	Mean	SD	Cut-off	Mean	SD	Cut-off
1:100	0.126	0.016	0.172	0.137	0.028	0.219
1:200	0.119	0.010	0.150	0.129	0.021	0.191
1:400	0.116	0.012	0.152	0.118	0.017	0.169
1:800	0.107	0.008	0.131	0.109	0.013	0.148
1:1600	0.102	0.008	0.126	0.106	0.019	0.162
1:3200	0.104	0.027	0.184	0.104	0.011	0.137
1:6400	0.100	0.007	0.120	0.101	0.010	0.132
1:12,800	0.100	0.007	0.120	0.102	0.009	0.129
1:25,600	0.096	0.007	0.117	0.100	0.010	0.130
1:51,200	0.095	0.007	0.114	0.100	0.009	0.128
1:102,400	0.096	0.007	0.118	0.101	0.010	0.130
1:20	0.170	0.040	0.291	0.166	0.066	0.365

Note: Data are expressed as optical density (OD) values. *Abbreviation*: SD: standard deviation. Cut-off = mean OD + 3 SD.

### 2.7. ELISA Assay Validation

The analytical performance of the in-house ELISAs for anti-agalsidase α and β antibodies was evaluated in terms of sensitivity, specificity, precision, matrix effects and drug tolerance. Positivity cut-offs were calculated for each serum dilution using healthy, drug-naïve donors (n = 40), as the mean optical density (OD) plus three standard deviations (Table 2). Sensitivity was assessed through serial dilutions of ADA-positive sera (n = 15), determining the lowest relative antibody level detectable above the cut-off. Intra-assay and inter-assay precision were evaluated using ADA-positive (n = 20) and ADA-negative (n = 30) samples, tested in triplicate within the same run or across independent sessions, respectively, and expressed as coefficients of variation (CV%). Specificity was investigated through competitive inhibition experiments using soluble agalsidase in ADA-positive sera (n = 15), calculating the percentage reduction in OD. Matrix effects were evaluated by comparing OD values obtained from equivalent amounts of ADA-positive serum in assay buffer and ADA-negative serum matrices (n = 10), expressing differences as percentage signal variation. Drug tolerance was assessed by testing ADA-positive sera, stratified by ADA titer in the presence of increasing concentrations of soluble agalsidase (α or β), identifying the highest concentration allowing ADA detection. In particular, ADA-positive patients with low (n = 6), medium (n = 6) and high (n = 7) titers were tested. All these assay parameters are shown in Table S2. Comparability of the two assays was suggested by the similar values of the previous described validation parameters and by Bland–Altman comparability test (mean bias= −0.002; 95% CI= −0.02–0.02, SD = 0.01 OD) performed on 88 sera (Figure S1).

### 2.8. Statistical Analysis

All the analysis were performed using GraphPad Prism 10.4.1 and JASP v. 0.97.1 when appropriate. Continuous variables were summarized as median and interquartile range (IQR), whereas categorical variables were reported as absolute frequencies and percentages. Comparisons between two independent groups were performed using the Mann–Whitney U test for continuous variables and Fisher’s exact test or the chi-square test, as appropriate, for categorical variables. Comparisons among more than two groups were performed using the Kruskal–Wallis test followed, when appropriate, by Dunn’s post hoc test. Correlations between continuous variables were assessed using Spearman’s rank correlation coefficient. Multivariable logistic regression analysis was performed to evaluate factors associated with ADA positivity, including age, male sex, predicted null-type mutation, treatment duration, normalized α-Gal A activity at diagnosis and ERT product as covariates. Results were expressed as adjusted odds ratios (aORs) with 95% confidence intervals (CIs). Model discrimination was evaluated using the area under the receiver operating characteristic curve (AUC), and Nagelkerke’s R^2^ was reported. Multivariable linear regression analysis was performed to evaluate factors associated with lyso-Gb3 levels, including age, male sex, predicted null-type mutation, treatment duration, ADA positivity and ERT product as covariates. Lyso-Gb3 values were log10-transformed before analysis, and results were reported as unstandardized regression coefficients (B) with 95% CIs. Specifically, the regression coefficient B represents the change in log10 -transformed Lyso-Gb3 levels associated with a one unit increase on the independent variables. This corresponds to a multiplicative change in unexpressed Lyso-Gb3 levels of 10^B^. Multicollinearity was assessed using variance inflation factors (VIFs). Normal residual distribution, homoscedasticity (residuals vs. predicted) and influential observations (Cook’s distance > 1) were also evaluated. A *p* value < 0.05 was considered statistically significant.

## 3. Results

### 3.1. Prevalence of ADA Positivity in Patients Treated with Agalsidase-α and -β and the Role of Sex

**Figure 1:**
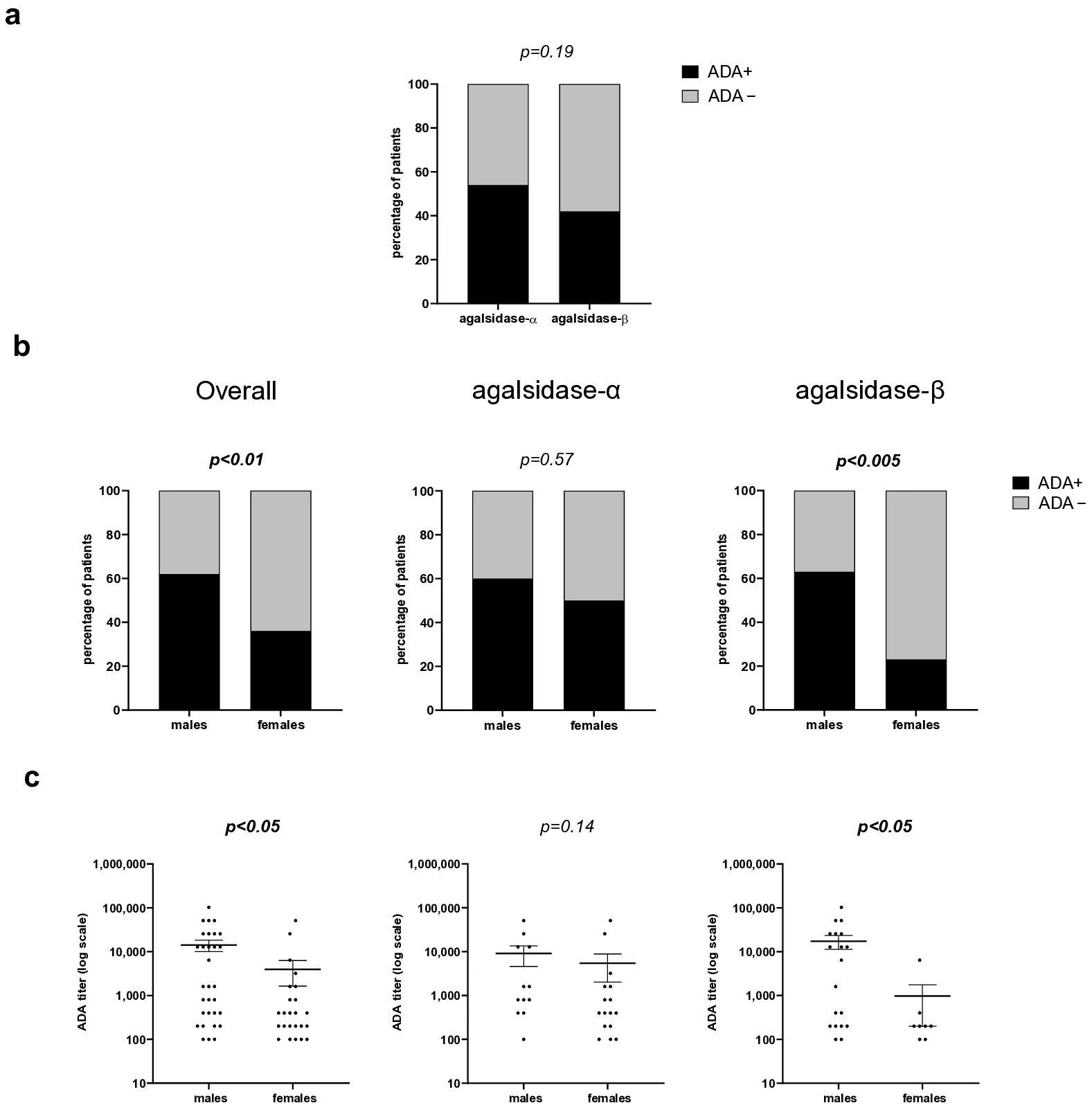
ADA prevalence and titers according to sex and enzyme replacement therapy. (**a**) Prevalence of ADA positivity in the overall cohort, in patients treated with agalsidase-alpha or agalsidase-beta. (**b**) ADA positivity according to sex in the overall cohort and after stratification by ERT. (**c**) ADA titers in ADA-positive male and female patients in the overall cohort and according to treatment with agalsidase-alpha or agalsidase-beta. *Abbreviation*: ADA, anti-drug antibody; ERT, enzyme replacement therapy.

Out of 117 patients tested for ADA, 62 (53%) were negative and 55 (47%) positive. As the patients analyzed were treated with agalsidase-α (52/117; 44.4%) or agalsidase-β (65/117; 55.6%), the analysis was performed on these two groups of patients. ADA positivity was compared between patients treated with agalsidase-α and agalsidase-β [28/52 (53.8%) vs. 27/65 (41.5%); 95% CI −5.8 to +30.4; *p* = 0. 19] (Figure 1a). The study was not powered to exclude a difference of this magnitude: with 52 and 65 patients the power to detect 60% versus 41.5% is 51%. Considering the possible impact of sex on ADA positivity, the prevalence of ADAs was accordingly analyzed. A statistically significant difference was observed, in that 31 out of 50 male (62%) and 24 out of 67 female (35.8%) patients were ADA-positive (*p* < 0.01) (Figure 1b left panel). By dividing patients in the two treatment regimens, no statistically significant difference between ADA+ male and female patients treated with agalsidase-α was observed (Figure 1b central panel). Whereas, regarding agalsidase-β, a statistically significant percentage of male patients was ADA-positive in comparison to females [19/30 (63.3%) vs. 8/35 (22.9%); *p* < 0.005] (Figure 1b right panel).

In terms of ADA-positive titers, as expected, a statistically significant difference was observed in males compared with females [1600 (IQR: 25,200) vs. 400 (IQR: 1200); *p* < 0.05] (Figure 1c left panel). This trend was also reported when the two drugs regimens were analyzed separately; the trend was observed in ADA levels for agalsidase-α even if it did not reach statistical significance (1200 (IQR: 12,300) in males vs. 400 (IQR: 1400) in females), while it was evident in agalsidase-β-treated patients [6400 (IQR: 25,400 vs. 200 (IQR: 225); *p* < 0.05] (Figure 1c, central and right panels). When we divided male and female patients into low (100–200), intermediate (400–800), high (1600–12,800) and very high (25,600–102,400) ADA titers (calculated by dividing ADA titers of positive patients in quartiles), we found that the majority of males had high and very high ADA titers while the majority of females showed low or intermediate ADA levels, although this comparison did not reach statistical significance (*p* = 0.132). The exact number of male and female patients included in each category are shown in Table S3.

### 3.2. Plasma Lyso-Gb3 Levels Are Higher in ADA-Positive Patients

**Figure 2:**
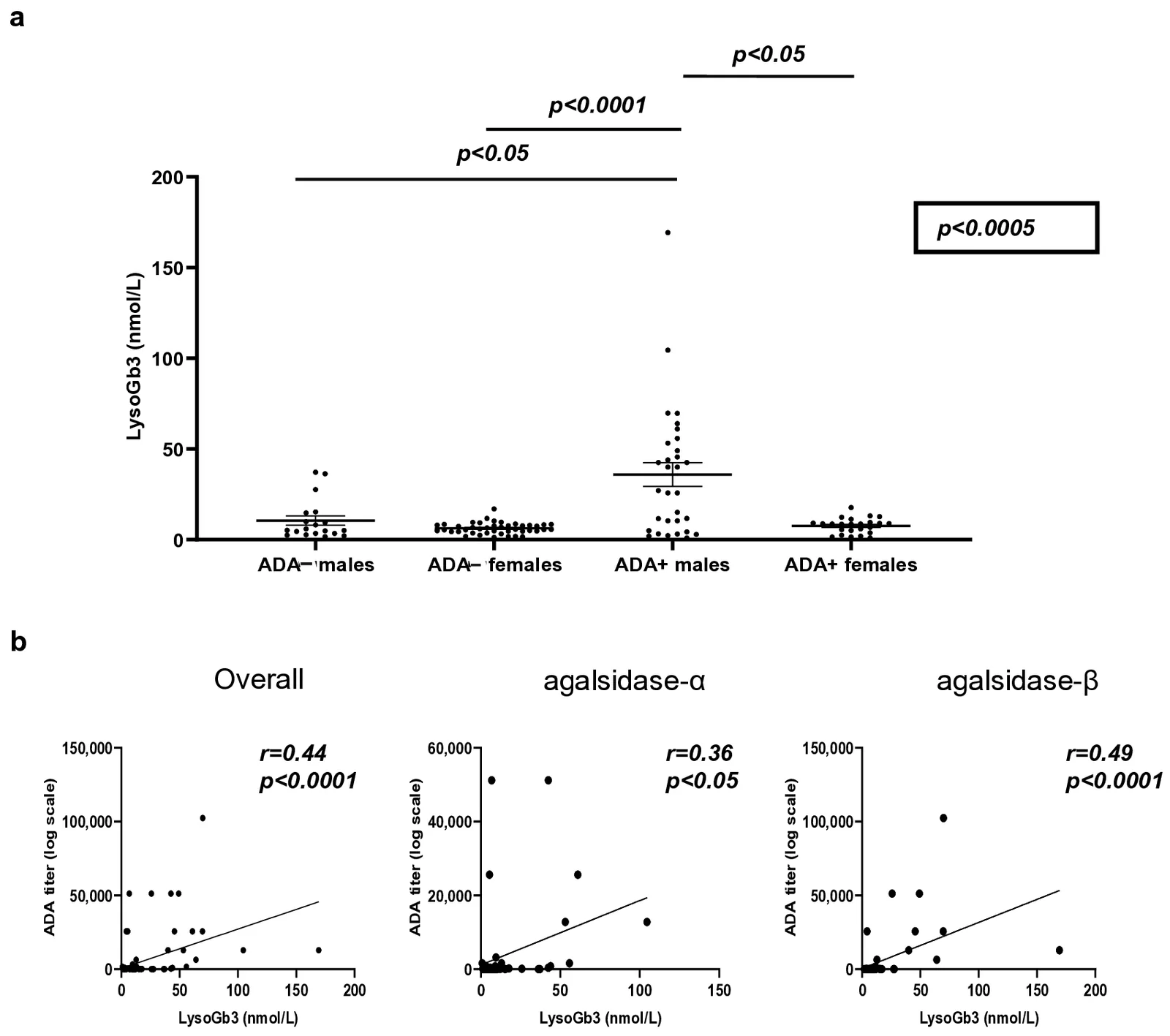
Association between ADA and circulating lyso-Gb3 levels. (**a**) Plasma lyso-Gb3 levels according to ADA status and sex in the overall cohort. (**b**) Correlation between ADA titer and plasma lyso-Gb3 levels in the overall population and separately analyzed in patients treated with agalsidase-alpha or agalsidase-beta. Correlations were assessed using Spearman’s rank correlation coefficient. *Abbreviation*: ADA, anti-drug antibody; lyso-Gb3, globotriaosylsphingosine.

On the collected samples of the 117 total patients, the Lyso-Gb3 plasma concentration was evaluated and related to serum ADA titers. As shown in Figure 2a, by analyzing ADA status and sex together, plasma Lyso-Gb3 levels differed significantly among the four groups (Kruskal–Wallis test, *p* < 0.0005). Post hoc multiple comparisons revealed that ADA-positive male patients had higher Lyso-Gb3 concentrations than ADA-negative male patients [27.1 (IQR: 48.3) vs. 5.2 (IQR: 11.3); *p* < 0.05) and female patients, regardless of ADA status. In female patients, by contrast, lyso-Gb3 did not differ significantly between ADA-positive and ADA-negative subjects [8.6 (IQR: 5.3) vs. 5.7 (IQR: 3.6) nmol/L; *p* = 0.1). In addition, in the overall population of our cohort there was a statistically significant positive correlation between ADA titer and plasma Lyso-Gb3 levels (r = 0.44; *p* < 0.0001) (Figure 2b, left panel). More importantly, for agalsidase-α-treated patients the correlation between ADA titer and serum Lyso-Gb3 levels was statistically significant (r = 0.36; *p* < 0.05) but more evident when agalsidase-β-treated patients were analyzed (r = 0.49; *p* < 0.0001) (Figure 2b, central and right panels).

### 3.3. Association of Predicted Null-Type Alleles with ADA Response and Lyso-Gb3 Levels

**Figure 3:**
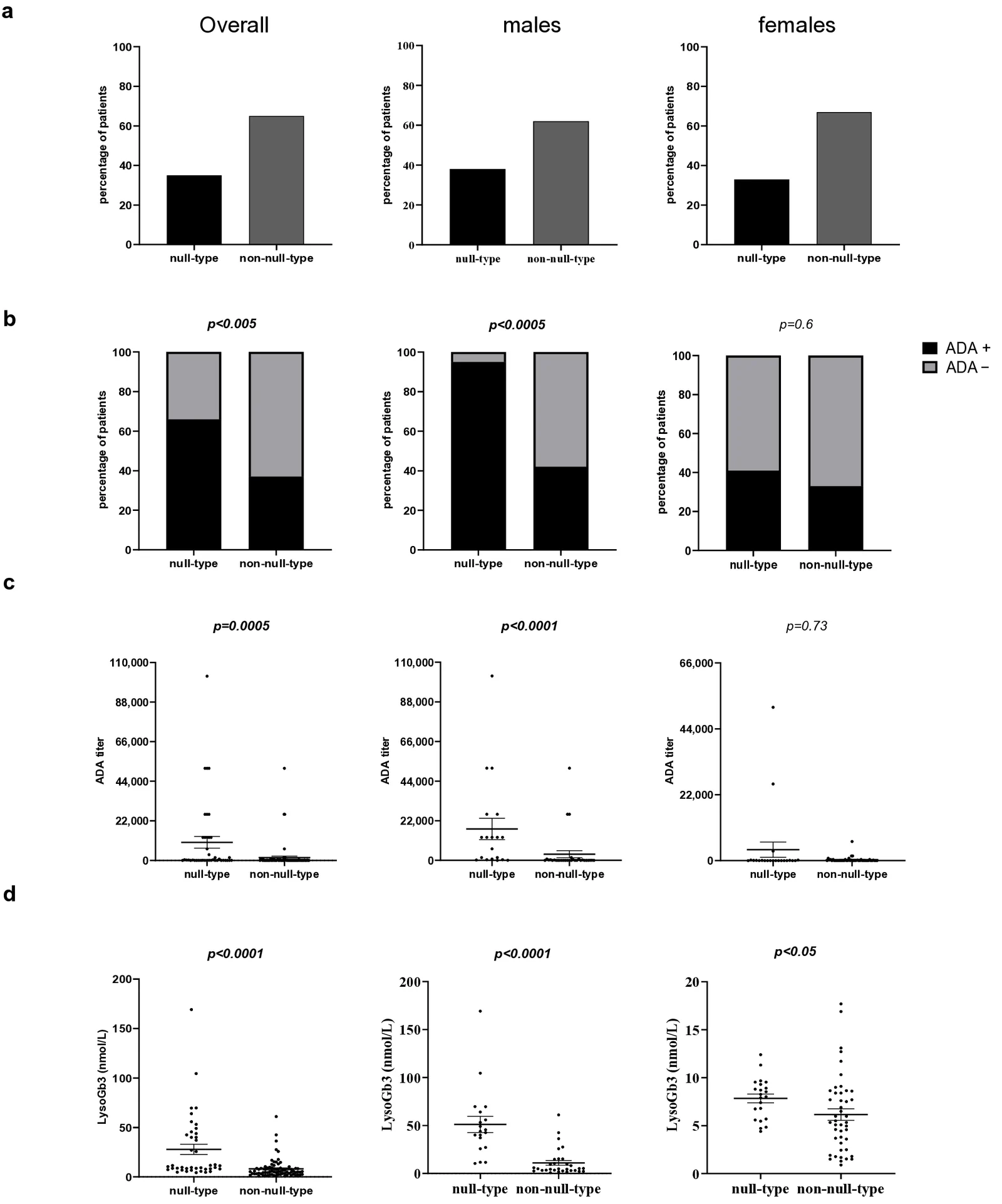
Association of predicted null-type GLA variants with ADA response and lyso-Gb3 levels. (**a**) Distribution of predicted null-type and non-null-type GLA variants in the study population. (**b**) Prevalence of ADA positivity according to allele category in the overall population and separately in males and females. (**c**) ADA titers according to allele category in the overall population and by sex. (**d**) Plasma lyso-Gb3 levels according to allele category in the overall population and by sex. *Abbreviation*: ADA, anti-drug antibody; GLA, galactosidase alpha; lyso-Gb3, globotriaosylsphingosine.

Overall, 41 patients (35.0%; 19 males and 22 females had a predicted null-type variant, whereas 76 (65.0%; 31 males and 45 females) had a predicted non-null-type variant (Figure 3a). When analyzed according to ADA status, patients with a predicted null-type allele showed a significantly higher frequency of ADAs compared with those with a predicted non-null-type variant (*p* < 0.005; Figure 3b, left panel). After stratification by sex, this difference was pronounced in males, with observable ADAs in 18/19 (95%) of males with a predicted null-type variant compared with 13/31 (42%) of males with a predicted non-null-type one (*p* < 0.0005). No significant difference was observed among females (Figure 3b, central and right panels).

Regarding ADA titers, patients with a predicted null-type variant showed significantly higher titers than those with a non-null-type variant [200 (IQR: 12,800) vs 0.0 (IQR: 200); *p* = 0.0005] (Figure 3c, left panel). This difference was more evident in males, in whom ADA titers were significantly higher in those with a predicted null-type variant than in those with a predicted non-null-type variant [12,800 (IQR: 25,200) vs. 0.0 (IQR: 400); *p* < 0.0001]. In females, no significant difference in ADA titers was observed between the two categories (*p* = 0.73; Figure 3c, central and right panels).

Finally, Lyso-Gb3 levels were significantly higher in patients with a predicted null-type variant than in those with a predicted non-null-type one [10.4 (IQR: 35.2) vs. 5.2 (IQR: 5.9) nmol/L; *p* < 0.0001] (Figure 3d, left panel). This difference remained significant in males [43.9 (IQR:36.9) vs. 4.9 (IQR: 11.8) nmol/L; *p* < 0.0001], as in females [8.1 (IQR: 3.7) vs. 5.3 (IQR: 5.7) nmol/L; *p* < 0.05] (Figure 3d, central and right panels).

### 3.4. ADA Positivity Is Related to GLA Variant in Male Patients

**Figure 4:**
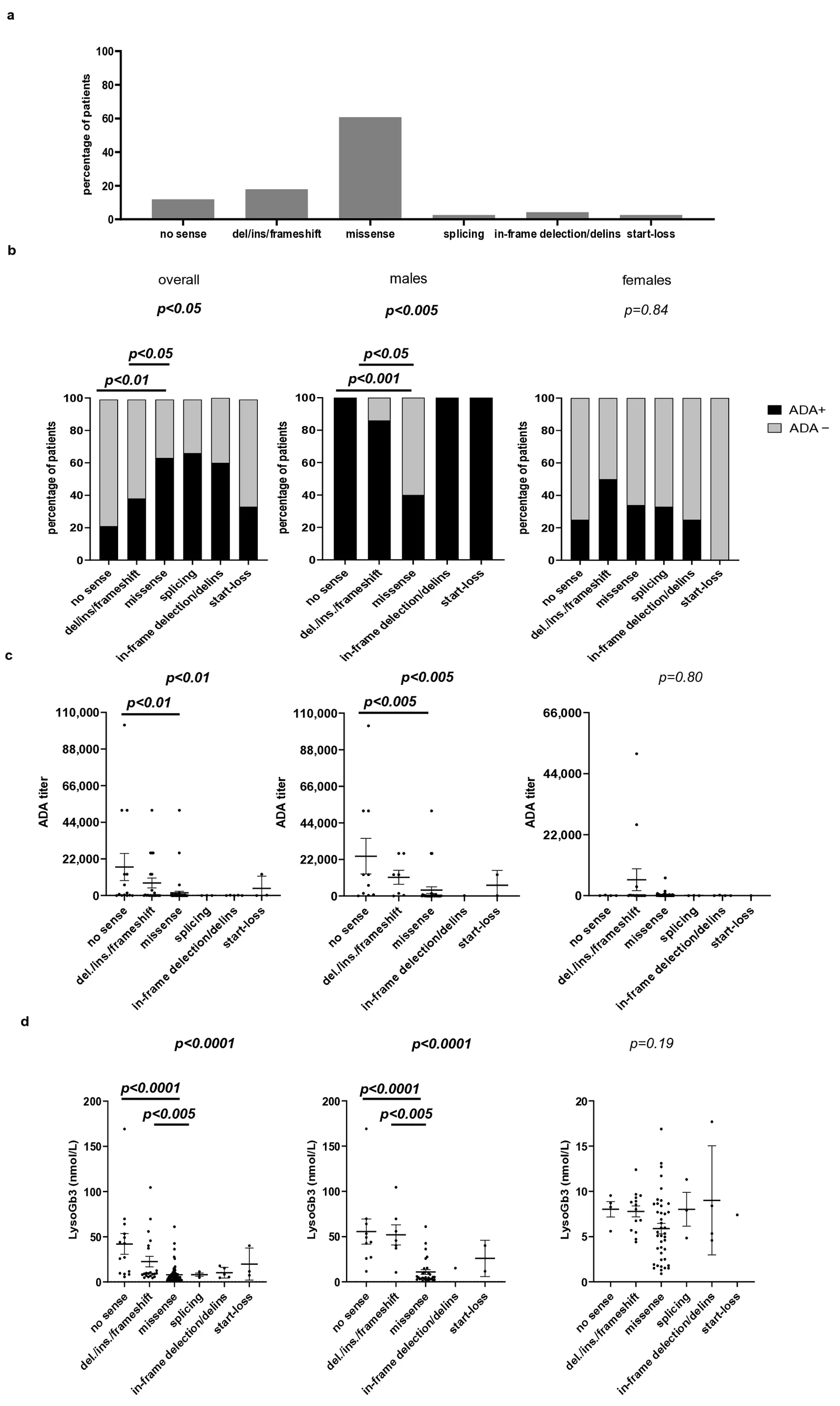
ADA response and plasma lyso-Gb3 levels according to GLA variant class. (**a**) Distribution of GLA variant classes in the study population. (**b**) Prevalence of ADA positivity according to variant class in the overall population and separately in males and females. (**c**) ADA titers according to variant class in the overall population and by sex. (**d**) Plasma lyso-Gb3 levels according to variant class in the overall population and by sex. Comparisons involving more than two groups were performed using the Kruskal–Wallis test followed, where appropriate, by Dunn’s post hoc test. *Abbreviation*: ADA, anti-drug antibody; GLA, galactosidase alpha; lyso-Gb3, globotriaosylsphingosine.

Of the 117 Fabry patients included in the study, 71 (60.7%) carried a missense variant, 21 (17.9%) a deletion, insertion or frameshift variant, 14 (11.9%) a nonsense variant, five (4.3%) an in-frame deletion or delins variant, three (2.6%) a canonical splice-site variant and three (2.6%) a start-loss variant in the GLA gene (Figure 4a, individual genotypes are listed in Table S1).

When stratifying patients according to ADA status, a significantly higher proportion of patients with nonsense variants were ADA-positive compared with those with missense variants (*p* < 0.005) (Figure 4b left panel). The proportion was also higher in comparison with patients with a canonical splice-site variant but the low number of this group did not allow for a statistical significance to be reached. The relationship between nonsense variants and ADA positivity remained significant when males were analyzed. In fact, individuals with nonsense variants developed ADA in a statistically higher percentage in comparison with those with missense variants (*p* < 0.005) (Figure 4b central panel). Conversely for females, no statistically significant differences in the proportion of ADA-positive patients between variants were found (Figure 4b, right panel). Furthermore, ADA titers significantly differed according to variant type (Kruskal–Wallis test, *p* < 0.005). Overall, patients with nonsense variants showed the highest ADA titers [1200 (IQR: 22,325)], followed by deletion/insertion/frameshift [100 (IQR: 12,800)] and start-loss variant [200 (IQR: 12,800)], followed by in-frame deletion or delins variants [0.0 (IQR: 200)] and missense variants [0.0 (IQR: 400)], whereas splicing variants displayed the lowest titers [0.0 (IQR: 100)]. Post hoc multiple comparisons showed a statistical difference between nonsense and missense mutations (*p* < 0.01) (Figure 4c left panel). Dividing by sex, only men showed a difference between mutation type groups and ADA status (Kruskal–Wallis test, *p* < 0.005) with a statistical difference between nonsense [9600 (IQR: 50,500)] and missense mutations [0.0 (IQR: 400)](*p* < 0.005) (Figure 4c central panel). No statistical difference was observed between these groups in female patients (Figure 4c, right panel).

Lyso-Gb3 levels differed significantly according to mutation type in the overall population (Kruskal–Wallis, *p* < 0.0001), with significantly higher levels in patients carrying nonsense [34.8 (IQR: 46.6) nmol/L; *p* < 0.0001], and deletion, insertion or frameshift variants [9.3 (IQR: 31.8) nmol/L; *p* < 0.005] compared with missense variants [5.1 (IQR: 5.9) nmol/L]. A similar pattern was observed in males (Kruskal–Wallis, *p* < 0.0001), with higher Lyso-Gb3 levels in patients carrying nonsense [46.5 (IQR: 38.7) nmol/L; *p* < 0.001] and deletion, insertion or frameshift variants [45.6 (IQR: 32.5) nmol/L; *p* < 0.005] compared with missense variants [4.9 (IQR: 8.6) nmol/L]. No significant differences were observed among mutation types in females (*p* = 0.19) (Figure 4d).

### 3.5. Residual α-Gal A Activity at Baseline and ADA Titer

**Figure 5:**
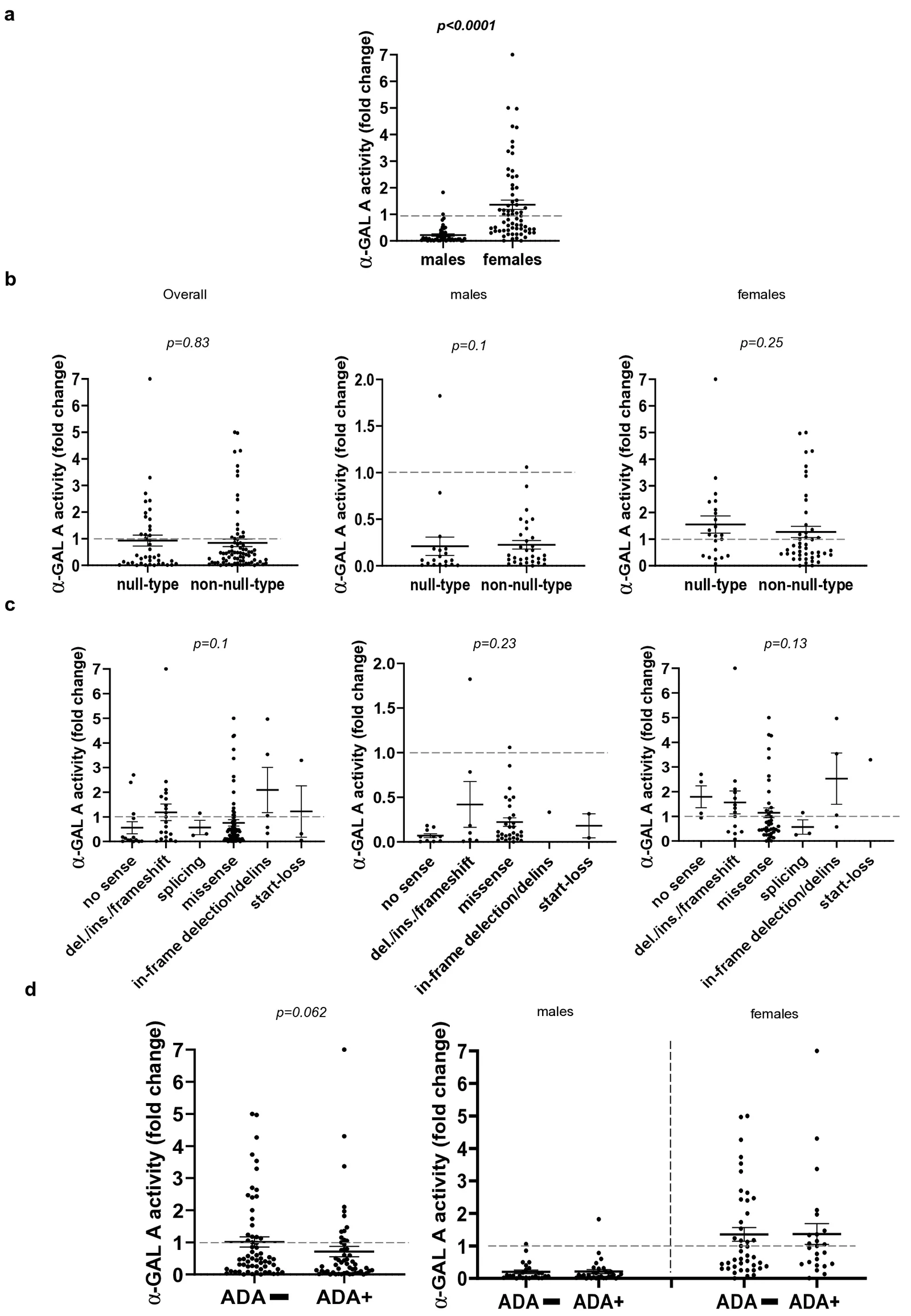
Residual α-galactosidase A activity according to sex, allele category, GLA variant class and anti-drug antibody status. (**a**) Baseline residual α-galactosidase A activity in male and female patients. (**b**) Residual α-galactosidase A activity according to predicted null-type or non-null-type variant category in the overall population and separately in males and females. (**c**) Residual α-galactosidase A activity according to GLA variant class in the overall population and by sex. (**d**) Residual α-galactosidase A activity according to ADA status in the overall population and separately in males and females. Enzyme activity was normalized to the lower limit of the reference interval of the assay used for each patient; a value of 1 (also represented by gray dotted line) represents the lower limit of normal. *Abbreviation*: ADA, anti-drug antibody; α-Gal A, α-galactosidase A; GLA, galactosidase-alpha.

Considering the importance of residual enzyme activity for immune tolerance toward ERT, baseline α-Gal A activity was analyzed in the whole cohort. Activity was expressed as fold change relative to the lower limit of the reference interval, with one representing normal activity (dashed line). Females showed significantly higher activity than males [0.8 (IQR: 1.6) vs. 0.1 (IQR: 0.2); *p* < 0.0001] (Figure 5a). Individual values, with the reference interval and the assay matrix of each measurement, are given in Table S1. Stratifying by allele category, residual activity was markedly lower in males than in females, regardless of allele category. Among males, residual activity was 0.1 (IQR: 0.2) and 0.1 (IQR: 0.3) for predicted null- and non-null-type variants, respectively (*p* = 0.1), whereas in females it was 1.2 (IQR: 1.8) and 0.7 (IQR: 1.0), respectively (*p* = 0.25) (Figure 5b).

Investigating each mutation category separately, α-Gal A activity was lowest among patients with nonsense variants in the overall population and in males, whereas in females it was lowest among carriers of in-frame deletion/insertion variants. However, no significant differences were observed among mutation categories in the overall population (*p* = 0.10), in males (*p* = 0.23), or in females (*p* = 0.13) (Figure 5c). The absence of a difference in females is consistent with random X-chromosome inactivation, which determines residual activity in heterozygotes largely independently of the variant carried on the affected allele.

Finally, when patients were stratified according to ADA status, α-Gal A activity tended to be lower in ADA-positive than in ADA-negative patients [0.3 (IQR: 0.9) vs. 0.5 (IQR: 1.0)], although the difference did not reach statistical significance (*p* = 0.062) (Figure 5d, left panel). By stratifying patients according to ADA positivity and sex, no statistically significant differences were found between ADA-positive and ADA-negative patients both among males and females (Figure 5d, right panel).

### 3.6. Sex, Predicted Null-Type Variants and Residual a-Gal Activity Were Linked to ADA Positivity in the Logistic Regression Analysis

**Figure 6:**
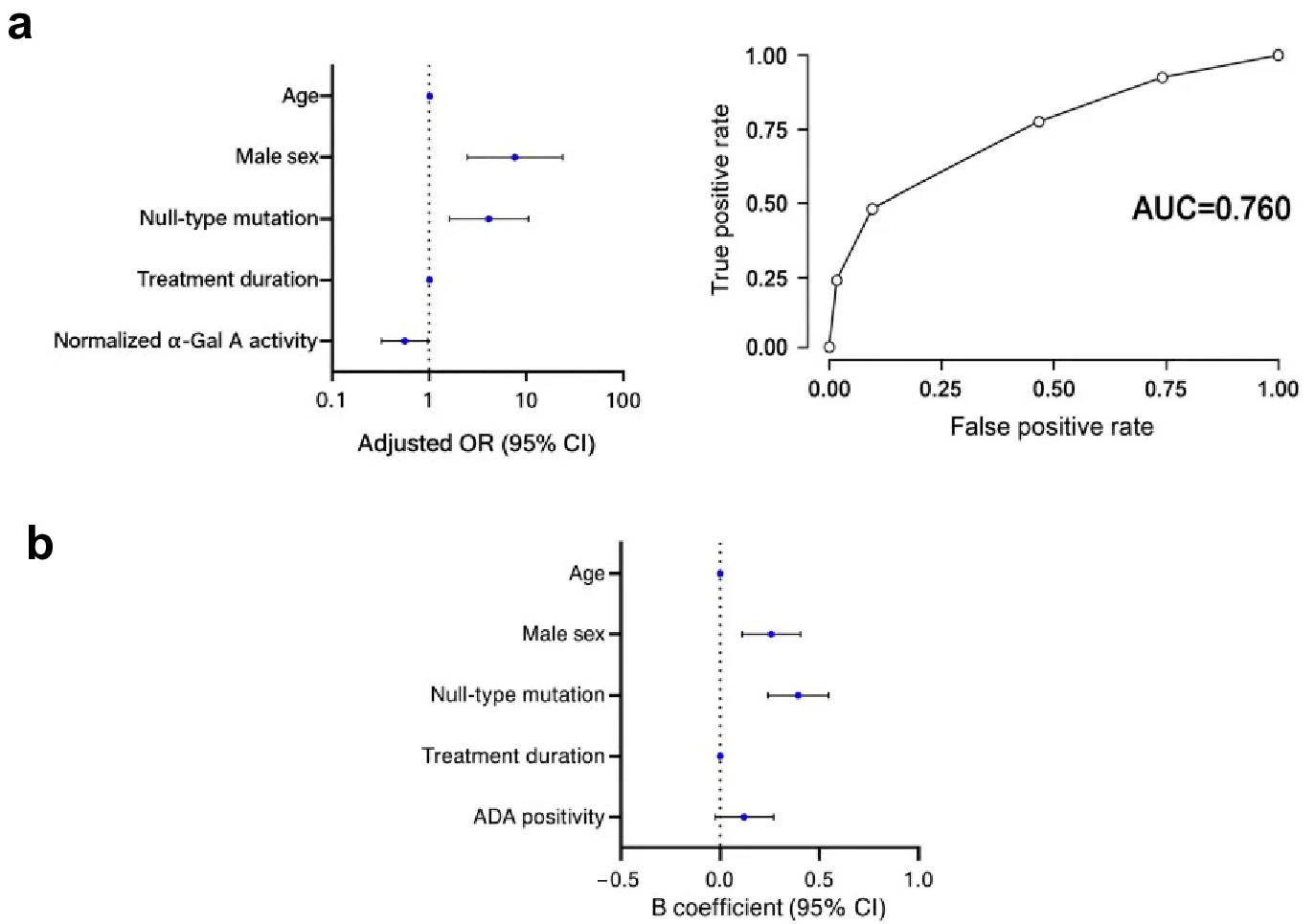
Multivariable analyses of factors associated with anti-drug antibody positivity and lyso-Gb3 levels. (**a**) Multivariable logistic regression analysis of factors associated with ADA positivity, with adjusted odds ratios and 95% confidence intervals, together with the receiver operating characteristic curve of the model. The model showed an area under the curve of 0.782. (**b**) Multivariable linear regression analysis of factors associated with circulating lyso-Gb3 levels. Lyso-Gb3 values were log10-transformed before analysis. Effect estimates are reported with 95% confidence intervals. *Abbreviation*: ADA, anti-drug antibody; AUC, area under the curve; CI, confidence interval; ERT: enzyme replacement therapy; lyso-Gb3, globotriaosylsphingosine; OR, odds ratio.

To define the relationship between ADA detection and the various potentially influencing factors, we performed a multivariable logistic regression analysis including, age, male sex, predicted null-type variant, treatment duration, lower normalized α-Gal A activity at diagnosis and ERT product. Male patients showed markedly higher odds of ADA positivity (aOR: 8.37, 95% CI 2.64–26.5; *p* < 0.001), as did patients with a predicted null-type variant (aOR: 4.36, 95% CI 1.70–11.16; *p* = 0.003). Conversely, higher normalized α-Gal A activity at diagnosis was associated with lower odds of ADA positivity (aOR: 0.55, 95% CI 0.32–0.96; *p* = 0.04). Age, ERT product and treatment duration were not significantly associated with ADA status (Figure 6a, left panel). The overall model was statistically significant (*p* < 0.001) and showed fair discriminatory ability (AUC 0.782), with no evidence of relevant multicollinearity among covariates (all VIF < 2) (Figure 6a, right panel; Table 3).

Finally, by stratifying our cohort in males and females and repeating the model separately; it remains statistically significant (*p* = 0.003, AUC = 0.813) in males but only predicted null-type mutations showed independence in predicting ADA positivity (*p* = 0.004, AUC = 0.791). Conversely, in females the model resulted statistically significant (*p* = 0.003, AUC = 0.789) and only normalized α-Gal A activity at baseline was an independent predictor of ADA positivity (Table S4). With 50 males, 19 of whom ADA-negative, and six covariates, the male model is at risk of overfitting.

### 3.7. ADA Positivity Was Not Independently Associated with Lyso-Gb3 Levels in a Regression Linear Model

In univariable analysis, ADA positivity was significantly associated with higher lyso-Gb3 levels. However, this association was no longer statistically significant after adjustment for potential confounders in the multivariable linear regression model (B = 0.117, 95% CI −0.034 to 0.268; *p* = 0.127). In contrast, male sex (B = 0.264, 95% CI 0.112–0.415; *p* < 0.001) and predicted null-type variants (B = 0.397, 95% CI 0.240–0.554; *p* < 0.001) remained independently associated with higher lyso-Gb3 levels, whereas age, treatment duration and ERT product were not significantly associated with the outcome (Table 4 and Figure 6b). The overall model was statistically significant (R^2^ = 0.396; *p* < 0.001), with no evidence of relevant multicollinearity (all VIF < 2). Residuals showed a normal distribution (mean = −1.9 × 10^−4^; SD = 1.005) both in histogram and Q-Q plot; they followed homoscedasticity with a visually homogeneous distribution in residuals vs. predicted plot and no influential observations (with Cook’s distance > 1) were obtained.

## 4. Discussion

The present study, performed in a real-world setting, suggests that ERT in FD patients is burdened by the development of ADAs potentially capable of interfering with the effectiveness of therapy and/or inducing adverse infusion reactions. This data takes on greater importance considering that in our experience almost 50% of patients developed ADA. The prevalence of ADA positivity we observed in females was higher than in previously published series. In our cohort 35.8% of females were ADA-positive, compared with 12.4% in the Fabry registry, 8% in the French FFABRY cohort, none in the Amsterdam cohort assessed by a neutralization assay [21] and in the randomized comparative trial of the two ERT preparations [22]. The prevalence in male patients was in the same order as in those series (Fabry French) [21]. However, some concerns may arise considering that these comparisons are descriptive, and the case series differ in assay, population and period of observation. Although our study was underpowered for this outcome, we observed no significant differences in terms of ADA production between patients treated with agalsidase-α or -b; further adequately powered studies are needed to confirm these findings.

These data are in contrast with several published data as well as post-marketing surveillance data, which report that approximately 70% of agalsidase-β-treated patients develop ADA, compared to 24% of those treated with agalsidase-α [23,24]. When the two preparations are compared within a single cohort and with a single assay, as in the present study and in the randomized trial cited above, the difference between them is not apparent. The same study also reported that the occurrence of antibodies was not associated with residual leucocyte α-Gal A activity, nor with the presence of a missense or nonsense variant, a conclusion that differs from the genotype association we observed and from the findings of the Fabry Registry and of the FFABRY cohort, in which antibodies developed preferentially in men carrying non-missense GLA variants [21]. Several factors seem to have a greater impact and among them, gender appears the critical one affecting the development of ADAs. Our data, in line with Mauhin and colleagues, indicate that detectable ADAs occur more frequently in males regardless of the molecule administered [25].

In agreement also are the data reported by van der Veen and colleagues, in which male patients develop ADAs more frequently in comparison to female patients [10]. The possible explanation for the higher incidence of ADA in Fabry male patients is a direct consequence of the disease’s mode of genetic transmission [26]. This is the rationale for the stratification by allele category adopted in our study. In fact, antibody formation is expected to be more frequent in hemizygous males with a predicted null-type variant, in whom no full-length endogenous enzyme is produced, and the infused protein is encountered by the immune system as a neoantigen. A hypothetically analogous dependence of the immune response on the endogenous expression of the deficient protein is established in Pompe disease, where cross-reactive immunologic material (CRIM) status is operationally defined and where CRIM-negative infants develop IgG earlier, at higher and more sustained titers, and have a poorer response to enzyme replacement therapy [27]. No equivalent assay or validated classification exists for Fabry disease, and cross-reactive material was not measured in our patients: the parallel is drawn here as a mechanistic hypothesis consistent with our genotype data, and not as a status assigned to this cohort. However, the behavior of female patients in terms of ADA production raises some points for discussion. In our case series of FD patients, we have observed that even a non-negligible percentage of females developed ADAs, without a significant difference between variant classes. Although females can be partially protected by the normal X-chromosome, a proportion of them can developed ADAs as a consequence of the normal X-chromosome inactivation due to the process of lyonization [26]. In agreement, the data observed in our case series regarding residual enzyme production was higher in female patients. In these female subjects, the final effect can be a low residual enzyme production favoring the ADA development like classical males.

The higher prevalence of ADA positivity we found in females deserves particular attention, because it is the single point on which our cohort departs from the literature. Two explanations must be considered and we cannot at present distinguish between them. The first is analytical: our assay detects binding IgG by direct ELISA and comprises only the screening tier of the standard ADA workflow and not neutralization assays, which detect a functionally defined and necessarily smaller subset of antibodies. The second explanation is biological: low-titer binding antibodies may be more frequent in treated heterozygous females than currently recognized, and would have gone undetected in series based on neutralization.

Since our analysis is a cross-sectional study with a retrospective nature, the different periods of ERT exposure among our patients could be a confounding factor. This seems to be irrelevant, however, by the fact that ADAs in FD patients appear within the first 3–6 months after initiating ERT and in most patients remain detectable up to 10 years [28,29].

Circulating ADAs can alter the pharmacokinetics and pharmacodynamics of administered enzymes, particularly based on their titers. It has been hypothesized that in patients with low ADA titers, drug concentrations enough to be effective can remain, whereas in patients with high titers, a relevant portion of the drug may be neutralized with loss of effectiveness of the ERT over time [30,31,32]. In our study, both neutralizing and inhibiting tests on cell lines as well as the correlation between the presence of ADAs and the clinical course in individual patients were not performed. Therefore, we have to consider that in our study was not possible to draw conclusions about what the long-term clinical impact of ADAs on disease progression might be.

A possible negative impact of ADAs on the clinical outcomes of FD has been described in the literature [13,33] and ADA titer has been previously correlated with the plasma level of Lyso-Gb3 [34,35]. In our cohort, this correlation was not confirmed by the multivariate analysis so we cannot state this with absolute confidence and wider cohorts will clarify the point unequivocally. In agreement with ADA positivity and male gender, higher titers have also been observed in these patients. In our cohort the highest antibody titers were found in the carriers of nonsense variants, although these account for only 11.9% of the patients. Our results could be compared with those showing that high anti-agalsidase-β IgG titers are associated with the presence of Gb3 deposits in the endothelial cells of dermal capillaries and with data demonstrating that ADAs to agalsidase-α are associated with a no significant reduction in plasma lyso-Gb3 levels [13,21,36]. In addition, it has been shown that ADA positivity with significantly high titers were associated with worse renal function, higher left ventricular mass and higher disease severity score [13,37]. Recently, a systematic review of the literature on ADA status and therapeutic effectiveness was published [33]. Although the papers show a trend toward negative effects of ADA-positive status on therapy, the causal association remains questionable. The authors note that none of the papers from which the data were collected was specifically designed to investigate this issue, and the extreme heterogeneity of the reported data undermines its reliability. Therefore, a prospective, dedicated study is suggested to clarify the issue [33].

The potential negative impact of ADAs in terms of efficacy and safety of biological treatment including ERT might raise the discussion of whether the immune system might be targeted pharmacologically, particularly in nonsense male patients, before starting ERT in order to reduce the immunogenicity of the protein. It is worth noting that Fabry patients with established ADAs treated with systemic steroids and immunosuppressants displayed a reduction in ADA titers, but after tapering the treatment ADA titers increased again [38]. Prevention of ADA formation with the use of immunosuppressive agents before starting ERT might be a reliable strategy, although some concerns have been raised for FD patients due to possible safety problems. However, this strategy appears to be supported by the data of Lenders et al. showing that male patients with classic FD phenotype, who started ERT after transplant in association with the corresponding immunosuppressive drugs, did not produce ADAs [38]. However, properly designed studies to address this issue are lacking and will be mandatory in the future.

We can identify some limitations of this research: (a) the antibody assay was not totally validated against the full tiered scheme—the positivity cut-off was derived from forty drug-naïve donors and no cross-reactivity analysis was performed between the two ERT products; (b) our study lacks of the detection of neutralizing activity of ADAs, and consequently the correlation between this important parameter and the biochemical and clinical outcomes of FD, and further analysis is necessary to empower this aspect of the study; (c) the unavailability of Lyso-Gb3 plasma levels at baseline before ERT; (d) the retrospective cross-sectional multicenter nature of the study did not allow for the randomization of the treatment and the treatment durations of agalsidase-α and -β were significantly different; (e) the absence of a detailed analysis relating ADA status to organ involvement; (f) baseline residual α-Gal A activity was measured in different laboratories using distinct analytical methods and biological matrices—although enzyme activity values were normalized to the lower limit of each laboratory’s reference interval, this approach does not ensure complete comparability between measurements; (g) multivariate models stratified by sex did not show the same independent variables in ADA prediction as the general model—this limitation should be considered for future studies in order to empower the predictive factors of ADA development after ERT both in males and females; (h) the null-type and non-null-type categories are predicted from the variant class and were not confirmed at protein or RNA level, and the absence of mature enzymes were not demonstrated for the individual variants.

In conclusion, our study confirms that ERT in Fabry patients is associated with the production of ADA in a high proportion of subjects, particularly male patients with predicted null-type variants. In addition, there is no significant difference in ADA incidence between patients treated with agalsidase-α and -β.

## Figures and Tables

**Table 3:** Multivariate logistic regression analysis of factors associated with ADA positivity.

Predictors	Adjusted OR	95% CI	*p* Value
Age	1.02	0.99–1.05	0.27
Male sex	8.37	2.64–26.5	**<0.001**
Predicted null-type variant	4.36	1.70–11.16	**0.003**
Treatment duration (months)	1.00	1.00–1.01	0.617
Normalized α-Gal A activity at diagnosis	0.55	0.32–0.96	**0.04**
ERT product (β)	1.71	0.69–4.23	0.249

Note: Global model: *p* < 0.001, AUC = 0.782, Nagelkerke R^2^ = 0.297. All VIF are <2. *Abbreviations*: AUC: Area under the curve; CI: Confidence interval; ERT: Enzyme replacement therapy; OR: Odds ratio; VIF: Variance inflation factor. Statistically significant *p* values are shown in bold.

**Table 4:** Multivariate linear regression analysis of factors associated with Lyso Gb3 levels.

Predictors	B	SE	95% CI for B	*p* Value
Age	−0.002	0.002	−0.007–0.003	0.40
Male sex	0.264	0.076	0.112–0.415	**<0.001**
Predicted null-type variant	0.397	0.079	0.240–0.554	**<0.001**
Treatment duration (months)	0.0004	0.0005	−0.0005–0.001	0.360
ADA positivity	0.117	0.076	−0.034–0.268	0.127
ERT product (β)	−0.036	0.075	−0.184–0.112	0.629

Note: Global model: R^2^ = 0.396, *p* < 0.001. All VIF are <2. B represents the unstandardized regression coefficient. Lyso-Gb3 values were log10-transformed before analysis. *Abbreviations*: CI: Confidence interval; ERT: Enzyme replacement therapy; SE: Standard error. Statistically significant *p* values are shown in bold.

## Data Availability

The data supporting the findings of this study are available from the corresponding author upon reasonable request. Individual patient data are reported in Table S1.

## Supplementary Materials

The following supporting information can be downloaded at https://www.mdpi.com/article/10.3390/cells15212015/s1. Table S1: Individual demographic, genetic and residual alpha-Gal A activity data of patients with Fabry disease; Table S2: ELISA assay validation parameters for agalsidase-α and -β ADAs; Table S3: Male and female Fabry patients stratified by ADA titer category; Table S4: Multivariate logistic regression analysis of factors associated with ADA positivity stratified by sex; Figure S1: Comparability between agalsidase-α and -β ELISA assay; Figure S1 legend.

## Abbreviations

The following abbreviations are used in this manuscript:

ACMG/AMP	American College of Medical Genetics and Genomics/Association for Molecular Pathology
ADA	Anti-drug antibody
α-Gal A	α-Galactosidase A
aOR	Adjusted odds ratio
AUC	Area under the receiver operating characteristic curve
BCA	Bicinchoninic acid
BSA	Bovine serum albumin
CHO	Chinese hamster ovary
CI	Confidence interval
CRIM	Cross-reactive immunologic material
ELISA	Enzyme-linked immunosorbent assay
ERT	Enzyme replacement therapy
FD	Fabry disease
Gb3	Globotriaosylceramide
GLA	Galactosidase alpha gene
HD	Healthy donor
HGVS	Human Genome Variation Society
IgE	Immunoglobulin E
IgG	Immunoglobulin G
IQR	Interquartile range
Lyso-Gb3	Globotriaosylsphingosine
OD	Optical density
PBS	Phosphate-buffered saline
PEG	Polyethylene glycol
RT	Room temperature
TMB	3,3′,5,5′-Tetramethylbenzidine
VIF	Variance inflation factor
