# Supplementary material for: Anti-Drug Antibodies in Patients with Fabry Disease Treated with Agalsidase-Alpha or -Beta: A Retrospective Italian Multicenter Study
Source: Cells. 2026 Nov 5;15(21):2015. doi: 10.3390/cells15212015 (PMC13761485; doi:10.3390/cells15212015)
Supplement: Supplementary file 1 [file cells-15-02015-s001.zip › Figure S1 legend.pdf]

Figure S1: Comparability between agalsidase-α and -β ELISA assay

Bland-Altman plot showing comparability between agalsidase-α and -β ELISA assay. Blue dotted line shows mean bias (-0.002) and black dotted lines show 95% CI (from -0.02 to 0.02)
