# Supplementary material for: Anti-Drug Antibodies in Patients with Fabry Disease Treated with Agalsidase-Alpha or -Beta: A Retrospective Italian Multicenter Study
Source: Cells. 2026 Nov 5;15(21):2015. doi: 10.3390/cells15212015 (PMC13761485; doi:10.3390/cells15212015)
Supplement: Supplementary file 1 [file cells-15-02015-s001.zip › Table S1.pdf]

Number	Age	Sex	GLA variant (as reported)	Variant class (reclassified)	Predicted variant category	alpha-Gal A activity	Normal range	Matrix	ERT product
**No sense**									
1	50	F	c.901C>T	no sense	null-type	7,2	>3 nmol/L/h	DBS	beta
2	54	F	c.1146 C>A	no sense	null-type	8,1	>3 nmol/L/h	DBS	beta
3	76	F	c.427G>A, c.621T>A	no sense	null-type	17,2	>15.3 nmol/L/h	DBS	beta
4	75	F	c.658C>T;p.(Arg220*)\	no sense	null-type	1,6	>1.7 nmol/L/h	DBS	beta
5	37	M	c.658c>T	no sense	null-type	0,5	>3 nmol/L/h	DBS	alpha
6	27	M	c.901C>T	no sense	null-type	0,4	>3 nmol/L/h	DBS	beta
7	44	M	c.1024C>T	no sense	null-type	2,8	>15.3 nmol/L/h	DBS	alpha
8	43	M	c.658C>T	no sense	null-type	0,01	>3 nmol/L/h	DBS	beta
9	54	M	c658C>T; p.(ARG220*)	no sense	null-type	0,1	>1.7 nmol/L/h	DBS	beta
10	48	M	c.72 G>A	no sense	null-type	0,2	>15.3 nmol/L/h	DBS	beta
11	45	M	c.1095T>A;p.(Y365X)	no sense	null-type	1,83	30-200 nmol/mg prot/h	leucocyte	beta
12	43	M	c1095T>A; p.(Y365X)	no sense	null-type	1,83	30-200 nmol/mg prot/h	leucocyte	alpha
13	50	M	c658C>T; p.(ARG220*)	no sense	null-type	0,1	20-65 nmol/mg prot/h	leucocyte	beta
14	56	M	c.611 G>A, p.W204X	no sense	null-type	0,3	30-200 nmol/mg prot/h	leucocyte	beta
**Del./ins./frameshift**									
15	18	F	c.811delinsTCTCA;(p.Gly217Serfs*29)	del./ins./frameshift	null-type	0,9	>3 nmol/L/h	DBS	beta
16	64	F	p.(Leu414Cys fs Ter4)	del./ins./frameshift	null-type	5,2	>3 nmol/L/h	DBS	beta
17	56	F	c.1033_1034delTC	del./ins./frameshift	null-type	4,4	>3 nmol/L/h	DBS	alpha
18	27	F	c.994dupA	del./ins./frameshift	null-type	21	>3 nmol/L/h	DBS	alpha
19	36	F	c.718_719delAA	del./ins./frameshift	null-type	7,3	>3 nmol/L/h	DBS	beta
20	44	F	c.73delG	del./ins./frameshift	null-type	1,8	>3 nmol/L/h	DBS	alpha
21	63	F	c.1094dupA	del./ins./frameshift	null-type	3,5	>3 nmol/L/h	DBS	alpha
22	57	F	c.1033_1034delTC, p.S345Rfs*29	del./ins./frameshift	null-type	6,3	>3 nmol/L/h	DBS	alpha
23	36	M	c.124_25delAT	del./ins./frameshift	null-type	12	>15.3 nmol/L/h	DBS	alpha
24	49	F	c.1033_1034delTC, p.S345Rfs*29	del./ins./frameshift	null-type	5,9	>3 nmol/L/h	DBS	alpha
25	50	M	c.428dup	del./ins./frameshift	null-type	2,8	>15.3 nmol/L/h	DBS	beta
26	58	M	10214InsT	del./ins./frameshift	null-type	3,1	>1.7 nmol/L/h	DBS	beta
27	38	F	c.1091-1092del(P.ser364Leufs*10)	del./ins./frameshift	null-type	7,5	20-65 nmol/mg prot/h	leucocyte	alpha
28	50	M	c618_619del	del./ins./frameshift	null-type	0,5	20-65 nmol/mg prot/h	leucocyte	alpha
29	55	F	c. 1277_1278	del./ins./frameshift	null-type	1,41	20-65 nmol/mg prot/h	leucocyte	beta
30	57	F	c. 599_560 AT p. (Met187Valfs*6	del./ins./frameshift	null-type	20	20-65 nmol/mg prot/h	leucocyte	beta
31	50	F	c.317_327del11, p.Leu106ProfsTer13	del./ins./frameshift	null-type	39,9	30-200 nmol/mg prot/h	leucocyte	beta
32	20	M	c.599560delATP(Met187valfs*6)	del./ins./frameshift	null-type	0,3	20-65 nmol/mg prot/h	leucocyte	alpha
33	14	M	c.317_327del11, p.Leu106ProfsTer13	del./ins./frameshift	null-type	0,2	30-200 nmol/mg prot/h	leucocyte	beta
34	42	M	GLA: c.(?delCT)	del./ins./frameshift	null-type	2	20-65 nmol/mg prot/h	leucocyte	beta
35	70	F	c.1091-1092del(P.ser364Leufs*10)	del./ins./frameshift	null-type	7,5	20-65 nmol/mg prot/h	leucocyte	alpha
**In-frame del./delins**									
36	45	F	c.1121_1123delAAG;(p.Lys374_Gly375delinsArg)	in-frame del./delins	non-null-type	10,6	>3 nmol/L/h	DBS	beta
37	56	F	c.1163_1165delTCC	in-frame del./delins	non-null-type	14,9	>3 nmol/L/h	DBS	beta
38	59	F	c.618_627 delinsA;p.(Tyr207-Trp209del)	in-frame del./delins	non-null-type	8,8	>15.3 nmol/L/h	DBS	beta
39	39	F	c.618_627 delinsA;p.(Tyr207-Trp209del)	in-frame del./delins	non-null-type	1	>3 nmol/L/h	DBS	beta
40	73	R	c.618_627 delinsA;p.(Tyr207-Trp209del)	in-frame del./delins	non-null-type	16	>15.3 nmol/L/h	DBS	alpha

**Missense**										
41	18	M	c.187T>C	missense	non-null-type	0,3	>2.67 nmol/L/h	DBS	alpha
42	57	F	c.187T>C	missense	non-null-type	11,5	>2.67 nmol/L/h	DBS	beta
43	60	M	c.1078G>A	missense	non-null-type	0,2	>1.2 nmol/L/h	DBS	beta
44	44	M	c.1078G>A;p.(Gly360Ser)	missense	non-null-type	0	>1.2 nmol/L/h	DBS	beta
45	45	F	c.1025G>A;p.(Arg342Gln)	missense	non-null-type	15	>15.3 nmol/L/h	DBS	alpha
46	78	F	c.950T>C;p.(Ile317Thr)	missense	non-null-type	7,3	>15.3 nmol/L/h	DBS	alpha
47	45	F	p.(Ile232Thr)	missense	non-null-type	6,8	>15.3 nmol/L/h	DBS	beta
48	70	F	c.775C>T	missense	non-null-type	7,4	>15.3 nmol/L/h	DBS	alpha
49	44	F	c.486G>T;p.(Trp162Cys)	missense	non-null-type	10	>15.3 nmol/L/h	DBS	beta
50	68	M	c.644A>G;p.(Asn215Ser)	missense	non-null-type	1,2	>15.3 nmol/L/h	DBS	alpha
51	26	M	c.644A>G;p.(Asn215Ser)	missense	non-null-type	1,2	>15.3 nmol/L/h	DBS	beta
52	55	F	c.1066C>T;p.(Arg356Trp)	missense	non-null-type	12,8	>15.3 nmol/L/h	DBS	alpha
53	46	M	c.950T>C;p.(Ile317Thr)	missense	non-null-type	0,3	>15.3 nmol/L/h	DBS	alpha
54	57	F	c.265G>A	missense	non-null-type	5,6	>15.3 nmol/L/h	DBS	alpha
55	65	F	c.680G>A;p.(Arg227Gln)	missense	non-null-type	6,9	>15.3 nmol/L/h	DBS	alpha
56	79	F	c.1025G>A;p.(Arg342Gln)	missense	non-null-type	11,6	>15.3 nmol/L/h	DBS	alpha
57	17	F	c.644A>G;p(asn215ser)	missense	non-null-type	9,1	>15.3 nmol/L/h	DBS	alpha
58	34	M	c.644A>G;p.(Asn215Ser)	missense	non-null-type	0,3	>15.3 nmol/L/h	DBS	alpha
59	59	M	c.644A>G;p(asn215ser)	missense	non-null-type	0,7	>15.3 nmol/L/h	DBS	alpha
60	53	F	Y86C	missense	non-null-type	7,74	>15.3 nmol/L/h	DBS	alpha
61	56	F	c.644A>G;p(asn215ser)	missense	non-null-type	3,8	>15.3 nmol/L/h	DBS	alpha
62	79	M	c.1066C>T;p.(Arg356Trp)	missense	non-null-type	1,7	>15.3 nmol/L/h	DBS	beta
63	81	M	N215S	missense	non-null-type	1,5	>3 nmol/L/h	DBS	beta
64	25	M	c.845C>T Thr282Ile	missense	non-null-type	0,2	>3 nmol/L/h	DBS	beta
65	52	M	c.644A>G;p.(Asn215Ser)	missense	non-null-type	1,2	>3 nmol/L/h	DBS	beta
66	76	M	c.644A>G;p.(Asn215Ser)	missense	non-null-type	0,5	>3 nmol/L/h	DBS	alpha
67	57	F	c.514 T>C p.Cys172Arg	missense	non-null-type	7,4	>3 nmol/L/h	DBS	beta
68	76	F	c.644A>G;p.(Asn215Ser)	missense	non-null-type	7,9	>3 nmol/L/h	DBS	beta
69	17	M	c.265G>A	missense	non-null-type	1,5	>3 nmol/L/h	DBS	beta
70	43	F	c.1066C>T;p.(Arg356Trp)	missense	non-null-type	0,3	>3 nmol/L/h	DBS	beta
71	37	F	c.704C>G	missense	non-null-type	12,8	>3 nmol/L/h	DBS	beta
72	70	F	c.515G>A	missense	non-null-type	15	>3 nmol/L/h	DBS	alpha
73	63	F	c.334C>T;p.(Arg12Cys)	missense	non-null-type	2,6	>1.7 nmol/L/h	DBS	alpha
74	56	F	Leu106His	missense	non-null-type	2,1	>1.7 nmol/L/h	DBS	alpha
75	57	F	c.901 C>G; p.R301G	missense	non-null-type	3	>3 nmol/L/h	DBS	beta
76	64	M	c.644A>G; p.(Asn215Ser)	missense	non-null-type	1,45	>1.7 nmol/L/h	DBS	alpha
77	75	F	c.1066C>T; (p.Arg356Trp)	missense	non-null-type	0,7	>1.7 nmol/L/h	DBS	beta
78	53	M	C.644 A > G p.N215S	missense	non-null-type	1,8	>1.7 nmol/L/h	DBS	beta
79	57	M	c.559A>G	missense	non-null-type	0,2	>3 nmol/L/h	DBS	beta
80	60	F	c.781 G>T	missense	non-null-type	1,4	>3 nmol/L/h	DBS	alpha
81	40	M	c.515G>A	missense	non-null-type	0,8	>3 nmol/L/h	DBS	alpha
82	52	F	c.644A>G	missense	non-null-type	6,2	>15.3 nmol/L/h	DBS	alpha
83	69	F	c.223C>G	missense	non-null-type	18	>15.3 nmol/L/h	DBS	beta

84	70	F	c.680G>A, p.R227Q	missense	non-null-type	11,2	>3 nmol/L/h	DBS	beta
85	56	F	c.824T>A	missense	non-null-type	1,6	>3 nmol/L/h	DBS	beta
86	38	F	c.159 C>A	missense	non-null-type	6	>3 nmol/L/h	DBS	beta
87	53	M	N53K	missense	non-null-type	0,1	>3 nmol/L/h	DBS	beta
88	55	M	c.1072G>A	missense	non-null-type	0,2	>3 nmol/L/h	DBS	beta
89	51	M	c.656T>C	missense	non-null-type	1,8	>3 nmol/L/h	DBS	beta
90	69	F	c.337T >C	missense	non-null-type	10,1	>3 nmol/L/h	DBS	beta
91	62	F	c.1088G>Ap.ARG363HES	missense	non-null-type	2,3	>1.7 nmol/L/h	DBS	beta
92	50	M	p.(Arg301Gln)	missense	non-null-type	0,8	>1.7 nmol/L/h	DBS	beta
93	48	M	c.337T>C	missense	non-null-type	0,6	>3 nmol/L/h	DBS	beta
94	74	M	c.901 C>G; p.R301G	missense	non-null-type	0,9	>3 nmol/L/h	DBS	beta
95	48	M	c.680G>A	missense	non-null-type	0,3	>3 nmol/L/h	DBS	alpha
96	78	F	c.902G>C;p.(Arg301Pro)	missense	non-null-type	0,01	>1.7 nmol/L/h	DBS	alpha
97	59	F	c790 G>T	missense	non-null-type	1,2	>3 nmol/L/h	DBS	beta
98	34	M	c.708G>C	missense	non-null-type	0,1	>3 nmol/L/h	DBS	alpha
99	30	F	c.677C>T;ARG301PRO	missense	non-null-type	6,1	20-65 nmol/mg prot/h	leucocyte	beta
100	50	F	c.1072G>A; p.Glu358Lys	missense	non-null-type	3,4	20-65 nmol/mg prot/h	leucocyte	beta
101	43	M	c.1079G>A	missense	non-null-type	1,7	20-65 nmol/mg prot/h	leucocyte	alpha
102	64	F	c. 227T>C	missense	non-null-type	5	20-65 nmol/mg prot/h	leucocyte	beta
103	64	F	c.227T>Cp.(met76thr)	missense	non-null-type	5	20-65 nmol/mg prot/h	leucocyte	alpha
104	71	M	c.1079G>A	missense	non-null-type	0,3	20-65 nmol/mg prot/h	leucocyte	alpha
105	55	F	R 301 P;IVS4-16g->a	missense	non-null-type	0,01	20-65 nmol/mg prot/h	leucocyte	beta
106	73	F	c.1079G>A	missense	non-null-type	21,5	20-65 nmol/mg prot/h	leucocyte	alpha
107	72	F	c.334C>T;p.(Arg112Cys)	missense	non-null-type	2,6	20-65 nmol/mg prot/h	leucocyte	beta
108	43	F	c.208A>G	missense	non-null-type	8,8	20-65 nmol/mg prot/h	leucocyte	alpha
109	65	M	c.644A>G; p.(Asn215Ser)	missense	non-null-type	4,2	20-65 nmol/mg prot/h	leucocyte	alpha
110	56	M	c.902G>C;p.(Arg301Pro)	missense	non-null-type	0,8	20-65 nmol/mg prot/h	leucocyte	alpha
111	55	F	c 1244T>G (pLeu415Arg)	missense	non-null-type	15,9	20-65 nmol/mg prot/h	leucocyte	alpha
**Start-loss**									
112	64	F	c.1A>G;p.(Met1Val)	start-loss	null-type	5,6	>1.7 nmol/L/h	DBS	alpha
113	45	M	c.1A>G;p.(Met1Val)	start-loss	null-type	0,9	20-65 nmol/mg prot/h	leucocyte	beta
114	70	M	c. 1A>G (p. M1V)	start-loss	null-type	6,3	20-65 nmol/mg prot/h	leucocyte	beta
**Splicing**									
115	50	F	c.635_639+8del	splicing	null-type	17,6	>15.3 nmol/L/h	DBS	alpha
116	41	F	c. 548-3-553	splicing	null-type	5	20-65 nmol/mg prot/h	leucocyte	alpha
117	35	F	c.1000-1G>A	splicing	null-type	6,2	20-65 nmol/mg prot/h	leucocyte	beta
