# Supplementary material for: Anti-Drug Antibodies in Patients with Fabry Disease Treated with Agalsidase-Alpha or -Beta: A Retrospective Italian Multicenter Study
Source: Cells. 2026 Nov 5;15(21):2015. doi: 10.3390/cells15212015 (PMC13761485; doi:10.3390/cells15212015)
Supplement: Supplementary file 1 [file cells-15-02015-s001.zip › Table S2.pdf]

Table S2 ELISA assay validation parameters for agalsidase-α and -β ADAs

ELISA parameters	Agalsidase-α	Agalsidase-β
Sensitivity (%)	6.25	6.25
Intra-assay precision (%)	5.3	4.9
Inter-assay precision (%)	6.0	6.3
Specificity (%)	84.1	86.9
Matrix effect (%)	12.9	13.4
Drug tolerance (μg/mL)		
- Low ADA titres	23.3	28.7
- Medium ADA titres	180	172
- High ADA titres	700	733.3

Note: low ADA titres (100-400), medium ADA titres (800-6400), high ADA titres (12800-102400)
Abbreviations: ADAs, Anti-drug antibodies
