# Supplementary material for: Anti-Drug Antibodies in Patients with Fabry Disease Treated with Agalsidase-Alpha or -Beta: A Retrospective Italian Multicenter Study
Source: Cells. 2026 Nov 5;15(21):2015. doi: 10.3390/cells15212015 (PMC13761485; doi:10.3390/cells15212015)
Supplement: Supplementary file 1 [file cells-15-02015-s001.zip › Table S3.pdf]

**Table S3**. Male and female Fabry patients stratified by ADA titre category

| ADA titre | Overall (n=55) | Males (n=31) | Females (n=24) |
|---|---|---|---|
| Low n° (%) | 18 (32.7) | 7 (22.6) | 11 (45.8) |
| Intermediate n° (%) | 14 (25.5) | 7 (22.6) | 7 (29.2) |
| High n° (%) | 13 (23.6) | 9 (29) | 4 (16.7) |
| Very-high n° (%) | 10 (18.2) | 8 (25.8) | 2 (8.3) |

Note: ADA titre categories were defined by dividing the overall ADA titres of positive patients into quartiles: low (100-200), intermediate (400-800), high (1600-12800), very-high (25600-102400). No statistically significant differences were observed between male and female patients ($p$ = 0.132).
Abbreviation: ADA**:** anti-drug antibodies.
