# Supplementary material for: Anti-Drug Antibodies in Patients with Fabry Disease Treated with Agalsidase-Alpha or -Beta: A Retrospective Italian Multicenter Study
Source: Cells. 2026 Nov 5;15(21):2015. doi: 10.3390/cells15212015 (PMC13761485; doi:10.3390/cells15212015)
Supplement: Supplementary file 1 [file cells-15-02015-s001.zip › Table S4.pdf]

Table S4 Multivariate logistic regression analysis of factors associated with ADA positivity stratified by sex

	Males			Females		
**Predictors**	**Adjusted OR**	**95% CI**	**p value**	**Adjusted OR**	**95% CI**	**p value**
Age	0.99	0.95-1.06	0.70	1.05	1.00-1.11	0.06
Predicted null-type variant	28.4	2.91-276.5	**0.004**	1.90	0.51-7.08	0.341
Treatment duration (months)	1.00	0.99-1.01	0.311	1.01	0.99-1.02	0.08
Normalized α-Gal A activity at diagnosis	0.37	0.02-7.16	0.514	0.57	0.32-0.99	**0.04**
ERT product (β)	0.45	0.08-2.63	0.376	0.40	0.12-1.34	0.138

Note: For males, global model: p=0.004, AUC=0.791, Nagelkerke R^2=0.319; for females: : p=0.003, AUC=0.789, Nagelkerke R^2=0.341. All VIF are <2.

Abbreviations: CI: Confidence Interval; ERT Enzyme Replacement Therapy; :OR: Odds ratio
